# Medical students’ attitudes toward socioscientific issues in female reproductive anatomy teaching: a cross-sectional study

**DOI:** 10.1186/s12909-026-09676-9

**Published:** 2026-06-16

**Authors:** Zhen-Zhen Kou, Jing Gu, Yan Wu, Miao Tian, Yi Li, Yun-Qing Li

**Affiliations:** 1 Department of Human Anatomy, Histology and Embryology, School of Basic Medical Sciences, The Fourth Military Medical University, Xi’an, Shaanxi China; 2 Department of Foreign Languages, School of Basic Medical Sciences, The Fourth Military Medical University, Xi’an, Shaanxi China; 3 Department of Medical Ethics, School of Basic Medical Sciences, The Fourth Military Medical University, Xi’an, Shaanxi China; 4 Department of Obstetrics and Gynecology, Tangdu Hospital, The Fourth Military Medical University, Xi’an, Shaanxi China

**Keywords:** Socioscientific issues, Anatomy education, Female reproductive system, Medical students, Artificial intelligence, Sentiment analysis

## Abstract

**Objectives:**

This study aimed to assess medical students’ attitudes toward integrating socioscientific issues (SSI) into female reproductive anatomy teaching across multiple academic years, using the novel case of China’s first uterus transplantation, and to apply artificial intelligence (AI)-based sentiment analysis to evaluate their emotional responses.

**Results:**

A cross-sectional study was conducted among 163 medical students across three academic cohorts (2022, 2023, and 2024–2025) at a medical university in China. Over 95% of students rated the SSI-based teaching positively. AI-based sentiment analysis revealed that attitudes toward ethical questions varied: for embarrassment in close-relative transplantation, no significant differences were found across cohorts (χ² = 5.78, df = 4, *p* = 0.216); for adoption as an alternative to childbirth, attitudes also did not differ significantly (χ² = 6.62, df = 4, *p* = 0.157). Mean sentiment scores for ovarian transplantation were neutral to slightly negative across all cohorts (2022: -0.31; 2023: -0.08; 2024–2025: -0.12, weighted average of -0.17 for 2024 and − 0.02 for 2025).

**Conclusions:**

SSI integration using a culturally relevant case was well-received by medical students. AI-based sentiment analysis proved a valuable tool for quantifying emotional responses. Attitudes toward ethical issues did not differ significantly across the three cohorts, suggesting a rationale for the sustained integration of SSI in preclinical curricula to foster ethical reasoning.

## Introduction

Medical education aims to cultivate not only clinical competence but also ethical reasoning and humanistic values, requiring critical thinking and moral sensitivity to address complex societal challenges [1, 2]. Socioscientific issues (SSI) encompass social challenges arising from scientific advancements that involve ethical, moral, and societal dimensions, such as gene editing and reproductive technologies [3, 4]. Integrating SSI into preclinical curricula helps students understand that medicine is embedded in social and ethical contexts [5, 6].

The female reproductive system is a foundational topic in preclinical anatomy education, involving not only structural knowledge but also complex social and ethical considerations. The case of China’s first live birth after uterus transplantation offers a compelling SSI that bridges anatomy with clinical, ethical, and sociocultural questions [7–9]. Unlike Western cases, this case carries unique cultural significance in China, where family lineage and traditional values around reproduction remain influential.

Despite growing interest in SSI-based pedagogy, quantitative assessments across cohorts remain rare; many studies have focused on qualitative outcomes [10] or single-cohort descriptive reports [2], and few have examined whether attitudes differ at the same stage of training. In the Chinese context, quantitative studies using culturally specific cases are scarce. Additionally, AI-based sentiment analysis—a tool for objectively quantifying emotional responses—has not been applied to SSI education research, representing a gap this study addresses [11]. Recent quantitative studies have begun to explore SSI in medical education [12, 13], but none have focused on reproductive anatomy with a culturally specific case combined with AI-based sentiment analysis.

This study addresses these gaps by: (1) quantitatively assessing medical students’ attitudes toward SSI integration in anatomy education across multiple cohorts (2), using a culturally relevant case—China’s first uterus transplantation—as the SSI focus (3), applying AI-based sentiment analysis, and (4) examining whether attitudes differ across cohorts at the same stage of training. To our knowledge, this study represents the first quantitative cross-cohort assessment of SSI attitudes in Chinese reproductive anatomy education, the first use of a culturally specific uterus transplantation case in this context, and the first application of AI-based sentiment analysis with human-AI agreement verification in SSI research.

## Methods

### Study design and setting

This study employed a descriptive cross-sectional design to explore medical students’ attitudes toward integrating SSI into female reproductive anatomy teaching. The study was conducted at a medical university in China across three academic cohorts: 2022, 2023, and 2024–2025 (with 2024 and 2025 combined due to the small sample size in 2025), as part of the anatomy course. Importantly, this is a cross-sectional comparison between independent cohorts of students at the same stage of training (third year of the eight-year program), with no control group and no repeated measurements over time. All comparisons are descriptive in nature and do not imply causality or longitudinal change.

### Participants and sampling

This study used a convenience sampling technique to select participants. Medical students who met the inclusion criteria and were enrolled in the anatomy course during the study period were invited to participate. The inclusion criteria were: (i) students enrolled in the eight-year medical program (integrated undergraduate and postgraduate studies), (ii) attendance at the SSI-based lesson on the female reproductive system, and (iii) completion of the online discussion and feedback survey. Students who were unwilling to provide informed consent were excluded.

A total of 172 students initially participated across the four calendar years (2022: *n* = 54; 2023: *n* = 66; 2024: *n* = 43; 2025: *n* = 9). Among these, 35 students did not provide valid responses to at least one of the three open‑ended questions (Q9 - Q11) and were excluded. The remaining 137 students were included in the final sentiment analysis, grouped by the cohort in which they took the course: 2022 (*n* = 38), 2023 (*n* = 47), and 2024‑2025 (*n* = 52; the 2024 and 2025 cohorts were combined due to the small sample size in 2025). No demographic data (age, gender) were collected.

### SSI-based teaching session

**Fig. 1:**
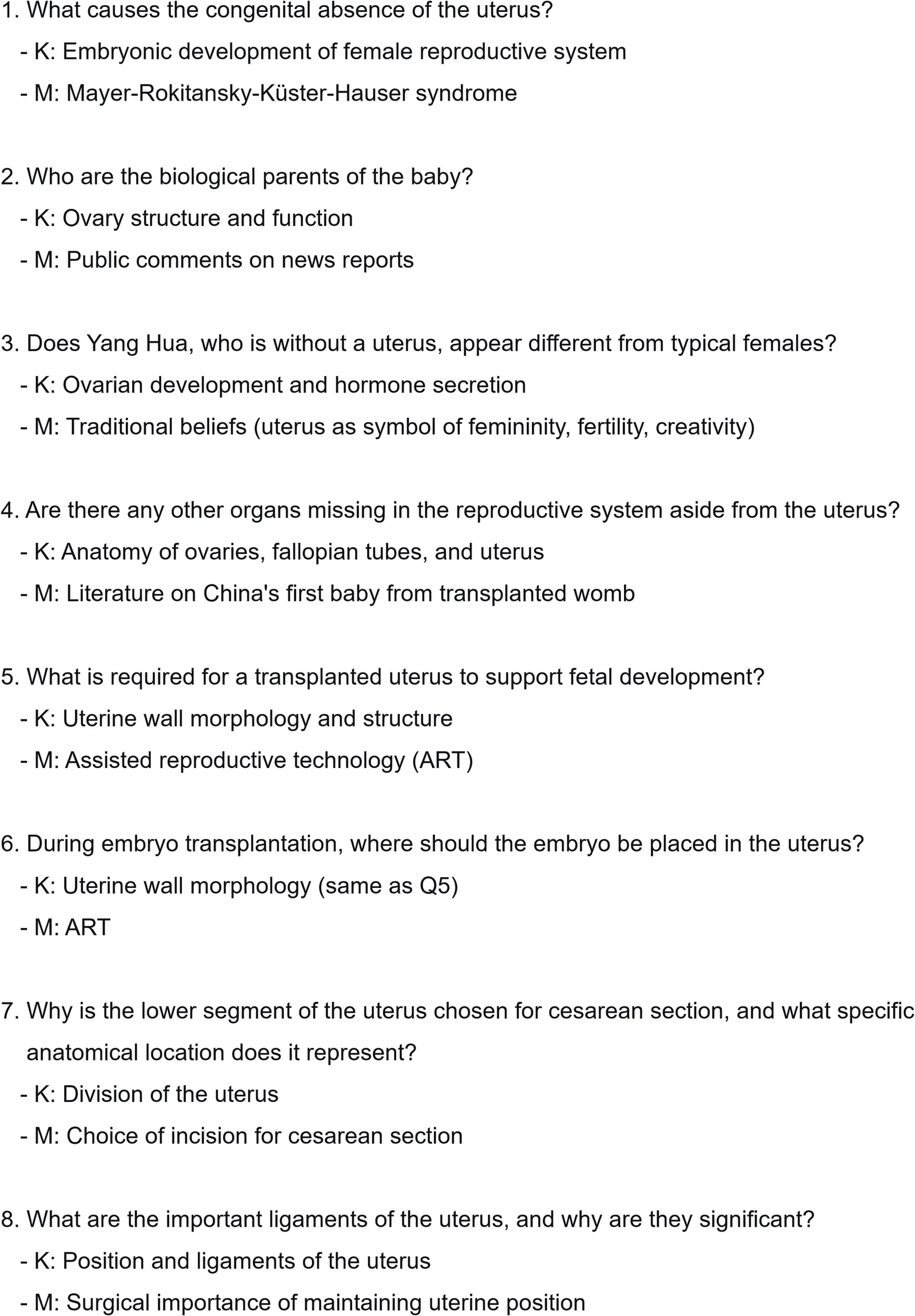
SSI case dicussion questions with corresponding knowledge points (K) and assistant materials (M)

The lesson was designed collaboratively by anatomy faculty, clinical obstetricians, and medical ethicists. The SSI case focused on China’s first uterus transplantation, involving a patient with congenital uterine absence who received a uterus from her mother and subsequently gave birth. The case was introduced during classroom instruction, followed by structured online discussions guided by eight questions addressing anatomical, clinical, and ethical dimensions (Fig. 1).

### Conceptual rationale and development of ethical discussion questions (Q9 - Q11)

The three open-ended ethical discussion questions (Q9 - Q11) were developed de novo by a multidisciplinary team consisting of anatomy faculty, clinical obstetricians, and medical ethicists. The questions were designed to capture distinct dimensions of students’ informal reasoning about SSI related to reproductive anatomy, guided by the SSI framework [14] and bioethical analyses of uterus transplantation [7–9]. Specifically, Q9 was intended to assess attitudes toward third‑party genetic material in reproductive technology; Q10 aimed to capture perceptions of social acceptability and embarrassment in intra‑familial organ donation; and Q11 was designed to explore preferences regarding biological lineage versus adoption, as well as cultural attitudes toward adoption.

### Data collection

After the lesson, students participated in an online discussion platform. Their responses to three key questions were analyzed:

1. Can a mother undergo transplantation of reproductive organs, including the ovaries, in cases of infertility? Why or why not?
2. Reproductive organ transplantation is a special type of organ transplantation. Is it embarrassing to perform such transplants within close relatives?
3. Can adoption serve as an alternative to natural childbirth? If you were the patient or her family member, how would you consider it?

Additionally, students completed a feedback survey on their perceptions of SSI integration, including eight closed-ended items rated on a 5-point Likert scale (1 = strongly agree, 5 = strongly disagree).

### AI-based sentiment analysis

Students’ responses to the three questions (Q9–Q11) were analyzed with an AI‑based sentiment analysis tool integrated into the online survey platform (www.wjx.cn). The tool uses a lexicon‑based Chinese natural language processing algorithm that assigns each answer a sentiment polarity (positive, neutral, or negative) and a continuous score from − 1 (most negative) to + 1 (most positive). Positive scores correspond to positive polarity, negative scores to negative polarity, and scores around zero (typically − 0.1 to 0.1) are classified as neutral.

For each question and cohort, the platform provided summary statistics, including mean sentiment scores and frequency distributions of sentiment categories.

To assess the accuracy of the AI classifications in our specific context (Chinese medical students’ ethical reasoning on reproductive technologies), two independent human raters blind to the AI output manually coded a random subset of 50 responses (approximately 36% of the total). The raters used the same three‑category scheme (positive/neutral/negative) following a brief training session. Agreement between the AI classification and the human consensus was 92% (46/50), and Cohen’s κ was 0.87 (95% CI: 0.78–0.96), indicating almost perfect agreement. This spot‑check supports the reliability of the AI classifications for the main analysis.

### Ethical considerations

This study was approved by the Ethics Committee of the First Affiliated Hospital of the Air Force Medical University (approval number: XJLL-KY-20262138) and adhered to the principles of the Declaration of Helsinki. The data were originally collected between 2022 and 2025 as part of routine teaching quality assurance activities; ethics approval was obtained retrospectively in 2026 for the analysis and publication of these anonymised data. Written informed consent was obtained from all eligible participants. Signed consent forms were stored securely and separately from the discussion data to maintain confidentiality. Confidentiality and anonymity were strictly maintained throughout the study by not collecting personal identifying information, coding all responses, and storing informed consent forms separately from the data.

### Statistical analysis

Data analysis was performed using SPSS version 26.0 (IBM Corp), incorporating descriptive and inferential statistics. Descriptive statistics included frequency, percentage, mean, and standard deviation.

For the closed-ended items (Q1 - Q8), due to the lack of variation (over 95% selecting the most positive option), inferential analysis was not conducted.

For the AI-based sentiment analysis, chi-square tests were used to compare sentiment category distributions across the three cohorts for Q9, Q10, and Q11, for which frequency data were available. Statistical significance was set at *p* < 0.05.

## Results

### Participant characteristics

Among the 172 initial participants, 35 were excluded because they did not provide valid responses to at least one of the three open-ended questions (Q9 - Q11). The final analysis therefore included 137 students: 38 from the 2022 cohort, 47 from the 2023 cohort, and 52 from the combined 2024–2025 cohort (2024: *n* = 43; 2025: *n* = 9).

### Student perceptions of SSI integration

Across all three academic cohorts, students rated the SSI-based teaching highly. Among the 172 initial participants, 163 completed the closed‑ended items (Q1 - Q8); the 2025 cohort (*n* = 9) only responded to the open‑ended questions (Q9 - Q11) and is therefore not included in Table 1. For the eight closed-ended items (Q1 - Q8), over 95% of students selected the most positive option (1 = strongly agree), indicating strong acceptance of the teaching approach. The remaining students selected the second most positive option. Table 1 presents the distribution of responses.

**Table 1:** Student perceptions of SSI integration (*n* = 163)

Item	Response 1 *n* (%)	Response 2 *n* (%)	Response 3–5 *n* (%)
Q1: Embryonic development demonstration	160 (98.2)	3 (1.8)	0 (0)
Q2: Understanding of anatomical structures	157 (96.3)	6 (3.7)	0 (0)
Q3: Introduction of MRKH syndrome	162 (99.4)	1 (0.6)	0 (0)
Q4: Integration of anatomy with MRKH	162 (99.4)	1 (0.6)	0 (0)
Q5: Quality of discussion questions	162 (99.4)	1 (0.6)	0 (0)
Q6: Recognition of SSI (uterus transplantation)	158 (96.9)	5 (3.1)	0 (0)
Q7: Discussion of scientific and ethical issues	162 (99.4)	1 (0.6)	0 (0)
Q8: Value of SSI for anatomy learning	162 (99.4)	1 (0.6)	0 (0)

Response options: 1 = strongly agree, 2 = agree, 3 = neutral, 4 = disagree, 5 = strongly disagree. Data are based on feedback questionnaires from 163 students (2022, 2023, and 2024 cohorts). The 2025 cohort (n = 9) participated only in the open-ended questions (Q9 - Q11) and did not complete the closed-ended items (Q1 - Q8); therefore they are not included in this table. The total number of initial participants was 172

### AI-based sentiment analysis of ethical questions

#### Q9: ovarian transplantation in infertility

The AI platform provided mean sentiment scores for each cohort: -0.31 (2022), -0.08 (2023), and − 0.12 (2024–2025; weighted average of -0.17 for 2024 and − 0.02 for 2025). Because individual holistic sentiment scores were not available, we approximated each student’s overall stance by averaging the sentiment scores of all opinions extracted from their textual response (available in the raw data export). Based on these per-student averages, the median (interquartile range, IQR) for Q9 were: -0.80 (IQR = 0.00) in 2022, -0.70 (IQR = 0.70) in 2023, and − 0.35 (IQR = 1.05) in the combined 2024–2025 cohort. The distribution of AI-classified sentiment categories (negative, neutral, positive) is presented in Table 2. A chi-square test revealed no statistically significant difference in the distribution of sentiment categories across the three cohorts (χ² = 8.46, df = 4, *p* = 0.076).

**Table 2:** AI sentiment categories for Q9 (ovarian transplantation in infertility)

Sentiment	2022 (*n*)	2023 (*n*)	2024–2025 (*n*)
Negative	11	12	17
Neutral	0	0	4
Positive	11	17	15
Total classified	22	29	36

Sentiment was classified by the AI tool integrated into the online survey platform (www.wjx.cn) as positive, neutral, or negative based on a lexicon‑based Chinese NLP algorithm. The table includes only responses that were successfully classified by the AI; some responses were unclassifiable (e.g., too short, ambiguous, or missing). Original cohort sizes: 2022, *n* = 38; 2023, *n* = 47; 2024 ‑ 2025, *n* = 52. Totals vary due to unclassifiable or missing responses for Q9

#### Q10: embarrassment in close-relative transplantation

The AI platform provided mean sentiment scores for each cohort: 0.31 (2022), 0.54 (2023), and 0.06 (2024–2025; weighted average of 0.00 for 2024 and 0.37 for 2025). Because individual holistic sentiment scores were not available, we approximated each student’s overall stance by averaging the sentiment scores of all opinions extracted from their textual response (available in the raw data export). Based on these per‑student averages, the median (IQR) for Q10 were: 0.55 (IQR = 0.70) in 2022, 0.70 (IQR = 0.40) in 2023, and 0.20 (IQR = 0.70) in the combined 2024–2025 cohort. The distribution of AI‑classified sentiment categories (negative, neutral, positive) is presented in Table 3. A chi‑square test revealed no statistically significant difference in the distribution of sentiment categories across the three cohorts (χ² = 5.78, df = 4, *p* = 0.216).

**Table 3:** AI sentiment categories for Q10 (embarrassment in close-relative transplantation)

Sentiment	2022 (*n*)	2023 (*n*)	2024–2025 (*n*)
Negative	13	9	19
Neutral	4	8	4
Positive	13	12	22
Total classified	30	29	45

Sentiment was classified by the AI tool integrated into the online survey platform (www.wjx.cn) as positive, neutral, or negative based on a lexicon‑based Chinese NLP algorithm. The table includes only responses that were successfully classified by the AI; some responses were unclassifiable (e.g., too short, ambiguous, or missing). Original cohort sizes: 2022, *n* = 38; 2023, *n* = 47; 2024‑2025, *n* = 52. Totals vary due to unclassifiable or missing responses for Q10

#### Q11: adoption as an alternative to childbirth

The AI platform provided mean sentiment scores for each cohort: 0.20 (2022), 0.04 (2023), and 0.13 (2024–2025; weighted average of 0.16 for 2024 and − 0.03 for 2025). Based on per-student averages, the median (IQR) for Q11 were: 0.47 (IQR = 0.80) in 2022, 0.20 (IQR = 0.80) in 2023, and 0.20 (IQR = 1.10) in the combined 2024–2025 cohort. The distribution of AI-classified sentiment categories is presented in Table 4. A chi-square test revealed no statistically significant difference across the three cohorts (χ² = 6.62, df = 4, *p* = 0.157).

**Table 4:** AI sentiment categories for Q11 (adoption as alternative to childbirth)

Sentiment	2022 (*n*)	2023 (*n*)	2024–2025 (*n*)
Negative	12	14	24
Neutral	1	1	3
Positive	16	14	14
Total classified	29	29	41

Sentiment was classified by the AI tool integrated into the online survey platform (www.wjx.cn) as positive, neutral, or negative based on a lexicon‑based Chinese NLP algorithm. The table includes only responses that were successfully classified by the AI; some responses were unclassifiable (e.g., too short, ambiguous, or missing). Original cohort sizes: 2022, *n* = 38; 2023, *n* = 47; 2024–2025, *n* = 52. Totals vary due to unclassifiable or missing responses for Q11

## Discussion

This study assessed medical students’ attitudes toward SSI integration in female reproductive anatomy teaching using AI‑based sentiment analysis. Students rated the SSI‑based teaching very positively (over 95% agreement), and attitudes toward three ethical questions did not differ significantly across cohorts. These findings are consistent with the feasibility of sustained SSI integration in preclinical curricula, providing empirical evidence that attitudes vary by topic but not by cohort, and suggesting that repeated exposure may be worth considering across training.

### Student acceptance of SSI-based teaching

The consistently high acceptance across cohorts aligns with previous reports that SSI increase student engagement by connecting science to real‑world dilemmas [15]. In anatomy education, embedding ethical discussions within structural content appears to help students appreciate the social and clinical relevance of basic science. The similar acceptance across cohorts suggests that this approach may be robust to cohort variation. This aligns with the broader SSI literature, which shows that engaging with real‑world dilemmas can foster students’ informal reasoning and use of evidence [13].

### Attitudes vary by topic, not by cohort

Students’ attitudes differed by topic but not by cohort. For ovarian transplantation (Q9), sentiment was balanced among negative, neutral, and positive categories. This mixed pattern likely reflects the ethical complexity of third‑party genetic material, where students weigh reproductive autonomy against concerns about genetic parenthood [16]. For close-relative transplantation (Q10), positive sentiment dominated, suggesting that students prioritize life-saving purpose over social discomfort - a finding consistent with the principle of beneficence in medical ethics. For adoption as an alternative to childbirth (Q11), attitudes were consistently divided. This division may stem from a combination of cultural emphasis on biological lineage, perceived social stigma around adoption, and concerns about the child’s welfare, as noted in previous research on reproductive attitudes in collectivist societies [10, 17]. Compared with findings from Ghana, where traditional and religious beliefs strongly shape attitudes toward assisted reproductive technology [10], our Chinese students also exhibited a cultural emphasis on biological lineage, but with less overt religious framing and more focus on filial piety and family continuity [17]. Importantly, the absence of cohort differences is consistent with the possibility that a single SSI lesson may not be associated with different deeply rooted ethical views, suggesting that repeated exposure may be important.

### Methodological contribution: AI-based sentiment analysis

The application of AI-based sentiment analysis enabled efficient, systematic classification of 137 open-ended responses with high reliability (92% agreement with human coding, κ = 0.87). This approach offers a practical tool for processing large-scale qualitative feedback in medical education research [18].

### Implications and need for sustained SSI integration

Our findings have three main implications. First, a culturally resonant case – China’s first uterus transplantation – may contribute to students’ engagement. Second, the absence of cohort differences precludes causal inferences but supports the interpretation that a single SSI session may not correlate with divergent ethical viewpoints. Whether repeated SSI exposure would lead to changes over time remains to be tested in longitudinal studies. Nevertheless, our findings support the consideration of integrating SSI repeatedly across multiple courses and years as a potential strategy to reinforce ethical reasoning. Third, AI-based sentiment analysis may be useful for educators to identify topics that generate strong or divided opinions, potentially informing targeted curriculum adjustments [12].

### Limitations

Several limitations should be acknowledged. First, the three ethical questions for discussion (Q9 - Q11) were ad-hoc and non-validated; findings should be interpreted as exploratory. Second, the cross-sectional design compares independent cohorts without a control group or repeated measurements, so the absence of differences does not imply intra-individual stability over time. Third, the single-institution sample limits generalizability, and demographic data (age, gender) were not collected. Fourth, although AI-based sentiment analysis showed high agreement with human coding (92%, κ = 0.87), the lexicon‑based algorithm, developed for general Chinese text, may misclassify culturally nuanced expressions, and is commercial/closed-source, limiting independent scrutiny. Despite these limitations, this study provides valuable preliminary evidence on SSI attitudes in anatomy education and demonstrates the feasibility of AI-based sentiment analysis in this context.

### Future directions

Further longitudinal research should be conducted to track ethical reasoning development of individual students. Multicenter studies would help assess generalizability across different institutional and cultural contexts. Collecting demographic data would allow for analysis of factors influencing attitudes. Comparing AI-based sentiment analysis with human coding could further validate this approach for medical education research. Additionally, exploring whether repeated SSI exposure across multiple courses influences attitude change would inform curriculum design.

## Data Availability

The data that support the findings of this study are available on request from the corresponding author.

## References

[1] Herman BC, Clough MP, Rao A. Socioscientific Issues Thinking and Action in the Midst of Science-in-the-Making. Sci Educ (Dordr). 2022;31(5):1105–39. 35035096 10.1007/s11191-021-00306-y PMC8741556

[2] Powell W. A socioscientific issues approach to ninth-graders’ understanding of COVID-19 on health, wealth, and educational attainments. PLoS ONE. 2023;18(3):e0280509. 36972262 10.1371/journal.pone.0280509 PMC10045461

[3] Sagmeister KJ, Schinagl CW, Kapelari S, Vrabl P. Students’ Experiences of Working With a Socio-Scientific Issues-Based Curriculum Unit Using Role-Playing to Negotiate Antibiotic Resistance. Front Microbiol. 2020;11:577501. 33552005 10.3389/fmicb.2020.577501 PMC7855855

[4] Keselman A, Levin DM, Hundal S, Kramer JF, Matzkin K, Dutcher G. Teaching Environmental Health Science for Informed Citizenship in the Science Classroom and Afterschool Clubs. Int J Sci Soc. 2012;3(3):31–44. 24382985 PMC3875328

[5] Westerhaus M, Finnegan A, Haidar M, Kleinman A, Mukherjee J, Farmer P. The necessity of social medicine in medical education. Acad Med. 2015;90(5):565–8. 25406609 10.1097/ACM.0000000000000571

[6] Zhang K. A New Scientific Medical Model: Improvement of the Current Biopsychosocial Medical Models. Res (Wash D C). 2024;7:0541. 10.34133/research.0541 PMC11612120 39628832

[7] Gao Y, Xue T, Chen B, Yang H, Wei L. Ethical issues raised by uterus transplantation: A report from the People’s Republic of China. Dev World Bioeth. 2023;23(1):34–40. 35187771 10.1111/dewb.12346

[8] Wei L, Xue T, Tao KS, Zhang G, Zhao GY, Yu SQ, et al. Modified human uterus transplantation using ovarian veins for venous drainage: the first report of surgically successful robotic-assisted uterus procurement and follow-up for 12 months. Fertil Steril. 2017;108(2):346–56. e1. 28778283 10.1016/j.fertnstert.2017.05.039

[9] Huang Y, Ding X, Chen B, Zhang G, Li A, Hua W, et al. Report of the first live birth after uterus transplantation in People’s Republic of China. Fertil Steril. 2020;114(5):1108–15. 33036792 10.1016/j.fertnstert.2020.06.007

[10] Asante-Afari K. Religious and cultural perspectives on assisted reproductive technology in Ghana: A comparative analysis of traditionalist, islamic, and christian beliefs. PLOS Glob Public Health. 2025;5(9):e0005240. 40996967 10.1371/journal.pgph.0005240 PMC12463212

[11] Busch F, Hoffmann L, Truhn D, Ortiz-Prado E, Makowski MR, Bressem KK, et al. Global cross-sectional student survey on AI in medical, dental, and veterinary education and practice at 192 faculties. BMC Med Educ. 2024;24(1):1066. 39342231 10.1186/s12909-024-06035-4 PMC11439199

[12] Ma I, Paget M, Desy J, Harvey A, Bendiak G, Naugler C, et al. A validity evaluation of lexicon-based sentiment analysis of medical students’ clinical performance from in-training evaluation reports. Med Educ. 2025. 10.1111/medu.70101. 10.1111/medu.70101 PMC13129623 41288118

[13] Jimenez P, Alred A, Dauer J. Describing undergraduate students’ reasoning and use of evidence during argumentation about socioscientific issues systems. Front Educ. 2024;9:1371095.

[14] Zeidler DL, Simmons ML, Howes EV, Beyond STS. A research–based framework for socioscientific issues education. Sci Educ. 2005;89(3):357–77.

[15] Schenk L, Hamza K, Arvanitis L, Lundegard I, Wojcik A, Haglund K. Socioscientific Issues in Science Education: An opportunity to Incorporate Education about Risk and Risk Analysis? Risk Anal. 2021;41(12):2209–19. 33960528 10.1111/risa.13737

[16] Salazar A, Diaz-Garcia C, Garcia-Velasco JA. Third-party reproduction: a treatment that grows with societal changes. Fertil Steril. 2023;120(3 Pt 1):494–505. 36681263 10.1016/j.fertnstert.2023.01.019

[17] Peng W. Impact of filial piety on residents’ subjective well-being in China considering the moderating effect of income level. Med (Baltim). 2024;103(3):e36983. 10.1097/MD.0000000000036983 PMC10798767 38241564

[18] Bhanvadia S, Radha Saseendrakumar B, Guo J, Spadafore M, Daniel M, Lander L, et al. Evaluation of bias and gender/racial concordance based on sentiment analysis of narrative evaluations of clinical clerkships using natural language processing. BMC Med Educ. 2024;24(1):295. 38491461 10.1186/s12909-024-05271-y PMC10944013

